# Intracellular Immunohistochemical Detection of Tetrodotoxin in *Pleurobranchaea maculata* (Gastropoda) and *Stylochoplana* sp. (Turbellaria)

**DOI:** 10.3390/md13020756

**Published:** 2015-01-28

**Authors:** Lauren R. Salvitti, Susanna A. Wood, Leigh Winsor, Stephen Craig Cary

**Affiliations:** 1 Department of Biological Sciences, University of Waikato, Private Bag 3105, Hamilton 3240, New Zealand; E-Mails: ls161@students.waikato.ac.nz (L.R.S.); susie.wood@cawthron.org.nz (S.A.W.); 2 Cawthron Institute, Nelson 7042, New Zealand; 3 College of Marine and Environmental Sciences, James Cook University, Townsville QLD 4811, Australia; E-Mail: leigh.winsor@jcu.edu.au

**Keywords:** *Pleurobranchaea maculata*, *Stylochoplana* sp., tetrodotoxin, immunohistochemistry, monoclonal antibody

## Abstract

Tetrodotoxin (TTX), is a potent neurotoxin targeting sodium channels that has been identified in multiple marine and terrestrial organisms. It was recently detected in the Opisthobranch *Pleurobranchaea maculata* and a Platyhelminthes *Stylochoplana* sp. from New Zealand. Knowledge on the distribution of TTX within these organisms is important to assist in elucidating the origin and ecological role of this toxin. Intracellular micro-distribution of TTX was investigated using a monoclonal antibody-based immunoenzymatic technique. Tetrodotoxin was strongly localized in neutral mucin cells and the basement membrane of the mantle, the oocytes and follicles of the gonad tissue, and in the digestive tissue of *P. maculata*. The ova and pharynx were the only two structures to contain TTX in *Stylochoplana* sp. Using liquid chromatography-mass spectrometry, TTX was identified in the larvae and eggs, but not the gelatinous egg cases of *P. maculata*. Tetrodotoxin was present in egg masses of *Stylochoplana* sp. These data suggest that TTX has a defensive function in adult *P. maculata*, who then invest this in their progeny for protection. Localization in the digestive tissue of *P. maculata* potentially indicates a dietary source of TTX. *Stylochoplana* sp. may use TTX in prey capture and for the protection of offspring.

## 1. Introduction

Tetrodotoxin (TTX) is a potent non-protein neurotoxin that selectively targets and blocks voltage-gated sodium channels. It is most notably found in the tissues of pufferfish species from the Tetraodontidae family [1,2]. Tetrodotoxin is fatal to humans (wt. 50 kg) at levels of just 1–2 mg [3]. Tetrodotoxin was initially thought to only occur in pufferfish, but has since been discovered in a growing number of organisms including frogs, newts, gastropods, crabs, an algae species, arrow worms, and land planarians [2,4,5,6].

Numerous researchers have suggested that the incidence of TTX in so many genetically unrelated organisms is due to an exogenous source such as symbiotic bacterial production or bioaccumulation through diet [2,7,8,9]. Bioaccumulation of TTX has been implicated in several instances in which prey animals have also been shown to contain TTX, or during captive studies where organisms removed from their natural environments lose their toxicity [1,10,11,12]. In contrast, bacterial production of TTX has also been reported in marine organisms including the gastropod *Niotha clathrata* [13], the blue-ringed octopus *Octopus maculosus* (*i.e.*, *Hapalochlaena maculosa*) [14], and the pufferfish *Fugu vermicularis vermicularis* [15]. However, concentrations of toxin produced by isolated bacterial strains are generally orders of magnitude lower than host organisms, suggesting bacterial production is unlikely to be the sole source of toxin [7,8,16]. In contrast, studies on terrestrial newts (*Taricha granulosa*) showed an increase in toxin concentrations when kept in captivity and the ability to regenerate TTX after the release of toxin through the skin, suggesting an endogenous source [17,18]. Currently the definitive origin of TTX remains debated, and strategies for acquisition most likely vary among species.

Studies using chemical methods to detect TTX have shown sequestration of toxin varies among tissue types in many organisms [2]. For example, in the pufferfish *Takifugu niphobles* high concentrations of TTX were present in the liver, ovaries, and intestines, while skin and muscle tissues only had low concentrations [1]. Micro-distribution of TTX has been demonstrated using TTX specific monoclonal antibody (mAB) immunoenzymatic techniques in newts [19,20], ribbon and flat worms [21], pufferfish [22,23,24], and octopuses [25]. Understanding the accumulation and sequestration of TTX at the cellular level provides additional information regarding the ecological functions of TTX. For example, in predator-prey trials conducted by Williams* et al.* [26] using the rough-skinned newts, *Taricha granulosa* and their natural predator the garter snake *Thamnophis sirtalis*, it was shown that rejected newts possessed significantly higher concentrations of TTX in the skin compared to those that were consumed.

In 2009, the opisthobranch *Pleurobranchaea maculata* was found to contain high concentrations of TTX when a number of dogs became ill after consuming beach-cast individuals in New Zealand [27]. Subsequent studies using liquid chromatography-mass spectrometry (LC-MS) revealed that only the TTX variant was present. The highest concentrations of TTX were in the mantle, gonad, and digestive tissue, with total TTX concentrations (highest average (ave.) 369 mg·kg^−1^) varying significantly between individuals and season [12,28]. Using a series of aquaria based studies, the egg-laying season was shown to coincide with seasonal peaks in TTX concentrations (June–August) [28]. The high concentrations of TTX detected in egg masses, and the subsequent depuration of TTX from adults after spawning, suggest that TTX plays a protective role in offspring of *P. maculata*. In 2013, high concentrations (ave. 376 mg·kg^−1^) of TTX were detected in *Stylochoplana* sp. (a marine flatworm), collected from Tauranga, New Zealand [29]. Concentrations of TTX were less variable than in *P. maculata*, but also decreased from winter (June–August) to spring (September to November). The small size of *Stylochoplana* sp. (*ca.* 60 mg) prohibited dissection and LC-MS analysis of TTX concentrations in various tissues, thus to date there is no information on how TTX is distributed within this organism. In this study immunohistological techniques, in conjunction with the T20G10 anti-TTX monoclonal antibody (mAB) [30], were used to investigate the micro-distributions of TTX within each organism at the cellular level. These data may provide insights on ecological function and the source of TTX in these organisms.

## 2. Results and Discussion

### 2.1. Pleurobranchaea Maculata

#### 2.1.1. Mantle

**Figure 1:**
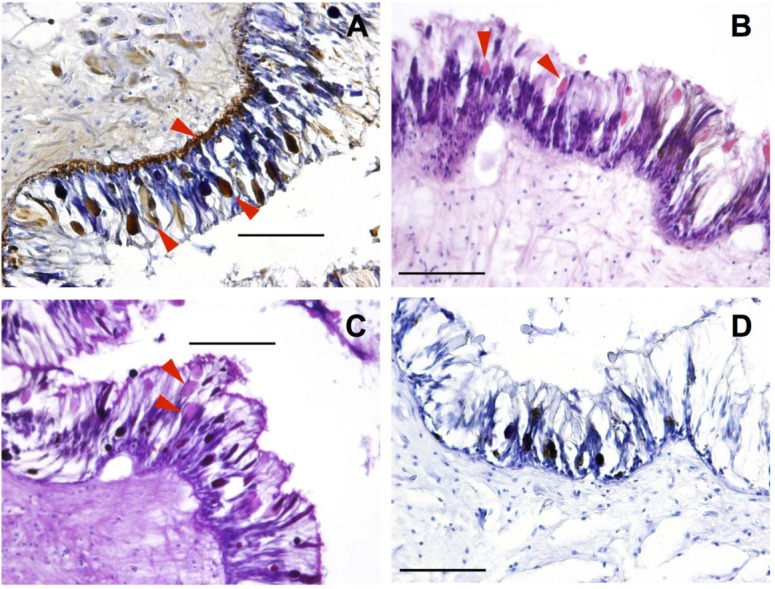
*Pleurobranchaea maculata* mantle tissue sectioned at 10 μm. Red arrows indicate tetrodotoxin (TTX) containing cells. (**A**) TTX-specific monoclonal antibody (mAB) immunohistological staining (TTX identified by the brown color deposits); (**B**) Hematoxylin and Eosin staining; (**C**) Alcian Blue-Periodic Acid Schiff staining and; (**D**) mAB negative control. Black bars = 100 μm.

Species from the group Opisthobranchia have extremely reduced, or in some cases have completely lost, their protective shell, resulting in a diverse range of alternative defensive strategies [31,32,33]. These include the acidification of the mantle [33,34,35], incorporation of nematocysts from cnidarian prey [31], development of spicules [36], secretion of ink [37], and acquisition of secondary metabolites [32,38]. The mantle, or dorsal body wall, of *P. maculata* consists of multiple folds or puckering of the epidermis which has previously been reported to be extremely acidic (pH = 1 to 2) [39]. In the immunostained section of the mantle TTX, visualized as brown color deposits, was most strongly localized in the basement membrane layer as well as in tear-shaped membrane bound cells (Figure 1A). This is similar to immunohistochemical studies on the pufferfish *Tetraodon nigroviridis* [22], *Tetraodon steindachneri* [24], and *Takifugu niphobles* [40] where TTX was shown to be sequestered in both basal cells and succiform cells of the epidermis. The pink color of the tear-shaped cells in both the Hematoxylin & Eosin (H&E) and the Alcian Blue–Periodic Acid Schiff (AB–PAS) stained sections reveal that these erythrophil cells secrete neutral mucin, suggesting that cells responsible for the acidity of the mantle and sequestration of TTX are separate. Sequestration of TTX in the skin has been reported in a number of other organisms including the pufferfish *Takifugu vermicularis* and *Chelonodon patoca*, [23], the California newt *Taricha torosa* [41], red-spotted newt, *Notophthalmus viridescens* [19], the Japanese newt *Cynops pyrrhogaster* [20], and the frog *Brachycephalus ephippium* [42], and a possible defensive mechanism is suggested.

#### 2.1.2. Reproductive and Digestive Tissue

**Figure 2:**
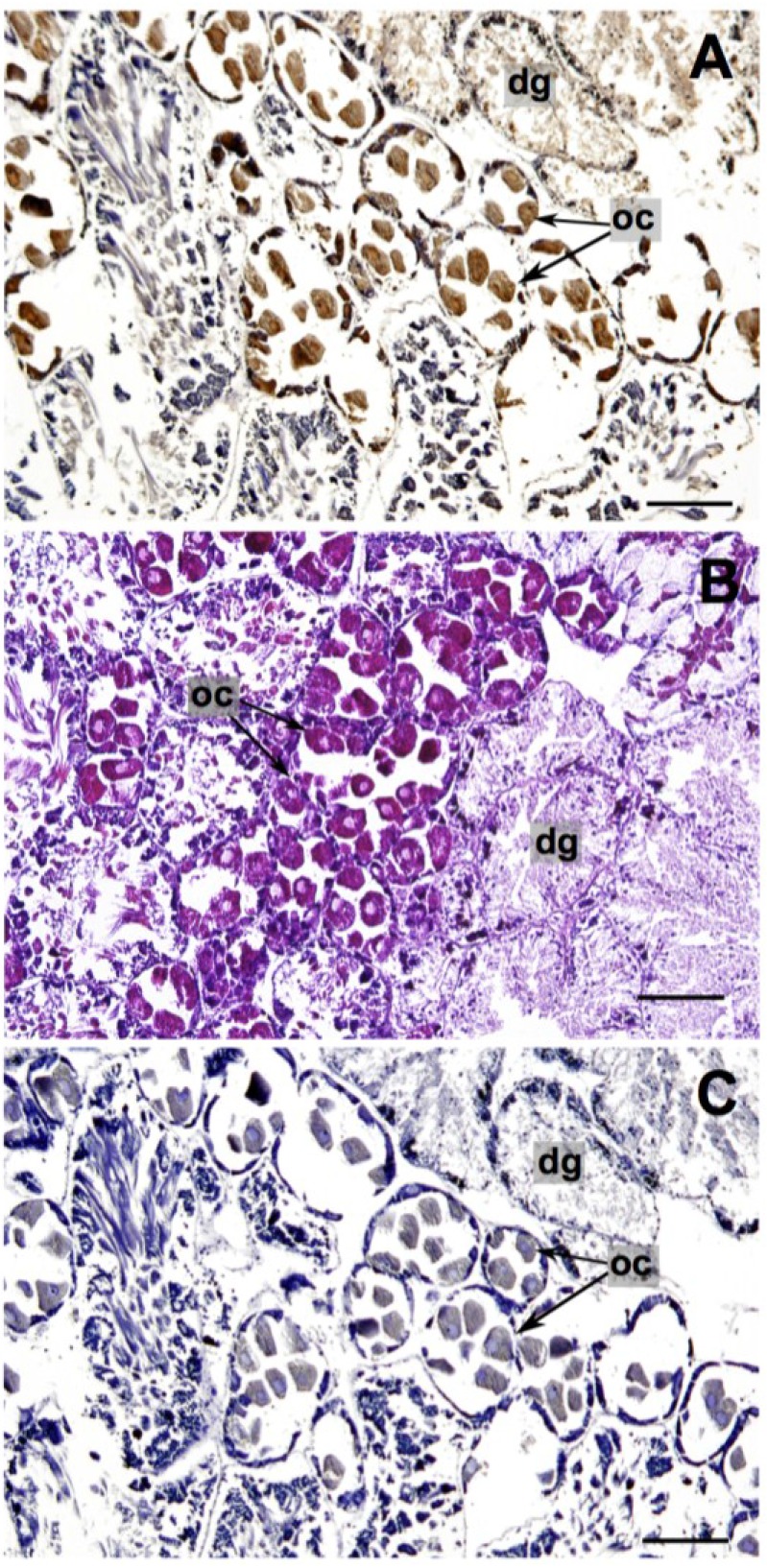
*Pleurobranchaea maculata* gonad/digestive tissue sectioned at 10 μm. (**A**) Tetrodotoxin (TTX)-specific monoclonal antibody (mAB) immunohistological staining (TTX identified by the brown color deposits); (**B**) Hematoxylin and Eosin staining; and (**C**) mAB negative control. dg = digestive gland, oc = oocyte. Black bars = 200 μm.

Analysis of the mAB incubation of the gonad and digestive tissue from *P. maculata* showed the strongest antigen-antibody reaction was in the oocytes and their surrounding follicles (Figure 2A). Positive staining occurred to a lesser degree in the digestive gland (Figure 2A). Localization of TTX in the reproductive organs/tissues has been observed in other organisms including the oocytes of pufferfish *Takifugu niphobles* [40], *Takifugu vermicularis* and *Chelonodon patoca* [23], the ovaries, oviduct, and testis of the short-tailed newt *Cynops ensicauda* [43], and the ovaries of the blue ringed octopuses *Hapalochlaena lunulata* and *Hapalochlaena fasciata* [44]. Researchers have suggested that TTX plays a protective role in host organisms, and the localization of TTX in reproductive organs imparts the toxin onto offspring to increase their survival rates [44,45].

Previous reports addressing the origin of TTX in *P. maculata* have provided evidence suggesting that a dietary source is probable [12,29,46]. A dietary source was suggested by Wood* et al.* [12] due to the depuration of TTX in *P. maculata* when kept in captivity and fed a non-toxic diet. Khor* et al.* [46] demonstrated that non-toxic *P. maculata* have the ability to sequester TTX into their tissues when fed an artificial toxic food source. Further evidence was provided when real-time PCR assays revealed *P. maculata* ingested the co-existing TTX-containing *Stylochoplana* sp. [29]. Collectively these studies suggest that TTX in *P. maculata* is most likely obtained from a dietary source. The localization of TTX in the digestive gland tissue of *P. maculata* supports this proposed scenario (Figure 2A).

### 2.2. Stylochoplana *sp.*

**Figure 3:**
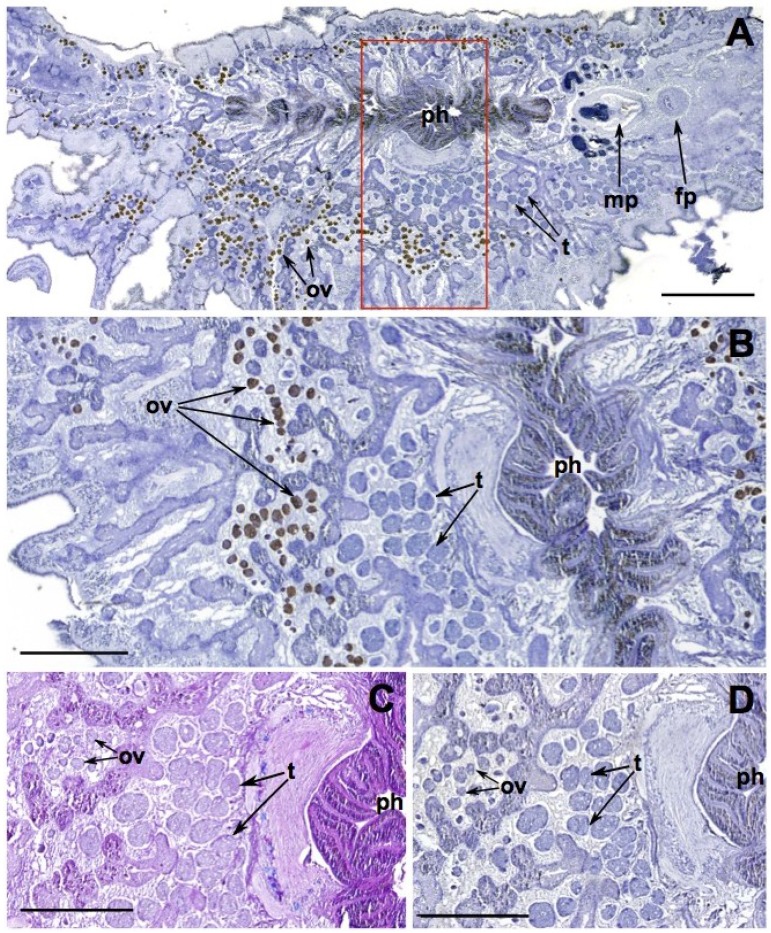
*Stylochoplana* sp. dorsoventral view sectioned at 7 μm. (**A**) Tetrodotoxin (TTX)-specific monoclonal antibody (mAB) immunohistological staining, (TTX identified by the brown color deposits) (**B**) Enlargement and 90° rotation of red box on A to show detailed view of ova, testes, and pharynx, (**C**) Alcian Blue-Periodic Acid Schiff staining, and, (**D**) mAB negative control. ph = pharynx, ov = ova, t = testes, mp = male pore, fp = female pore. Black bar = 1 mm (**A**), 500 μm (**B**–**D**).

*Stylochoplana* sp. were fixed flat and sectioned dorsoventrally. Tetrodotoxin, identified by the brown color deposits, is contained in the ova as well as portions of the pharynx (Figure 3). The localization of TTX in the reproductive and digestive tissues has also been reported in other flatworm species. Tanu* et al.* [21] used immunohistological techniques to show that TTX was contained in the ovum of the flatworm *Planocera reticulata*. Miyazawa* et al.* [47] showed the oviduct and digestive organs to be the most toxic tissues in the flatworm *Planocera multitentaculata* via mouse bioassay. A study on a planocerid species found on a reef in Guam demonstrated the highest concentrations of TTX were in the pharynx and through a series of feeding studies the researchers suggested that TTX was utilized in prey capture [48]. The sequestration of TTX in the pharynx of *Stylochoplana* sp. suggests this species may also use it to capture prey. The detection of TTX in the ova corroborates the observation of TTX in reproductive structures of *Pleurobranchaea maculata*, as well as many TTX-containing organisms, and most likely acts as a protective mechanism in offspring (see earlier discussion).

### 2.3. Offspring

The high water and mucopolysaccharide content of the gelatinous matrix of the egg masses prevented histological studies on these samples. However, the egg masses of both *P. maculata* and *Stylochoplana* sp., and hatched larvae of *P. maculata*, were tested for TTX utilizing liquid chromatography-mass spectrometry (LC-MS; Table 1). To ascertain where TTX is localized in the egg masses of *P. maculata*, the outer gelatinous matrix, with and without the eggs, was tested. No TTX was detected in the gelatinous matrix with the eggs removed, thus it is assumed that TTX is invested only into the eggs. While this would be beneficial for the larvae once hatched, the lack of TTX in the gelatinous coating surrounding the eggs is at odds with the suggestion that TTX provides a protective function for the egg masses [12]. This, in conjunction with the observation of the sea star *Patiriella regularis* consuming *Pleurobranchaea maculata* egg masses (L. Salvitti pers. obs.), suggests that TTX does not act as a predator deterrent at this life stage.

Tetrodotoxin concentrations of egg masses from a *Stylochoplana* sp. kept in aquaria were higher than those *P. maculata* egg masses tested in this study, but still in the range of previously recorded TTX concentrations of* P. maculata* egg masses (max. 100 mg·kg^−^^1^) [12]. It was not possible to localize TTX in egg masses of *Stylochoplana* sp. as these were only *ca.* 75 mg making separation of the gelatinous matrix and eggs difficult. Tetrodotoxin has been shown to be sequestered in eggs and egg masses of other species including flatworms *Planocera multitentaculata* [47], California newts *T. torosa* [41], rough skin newts *T. granulosa* [49], horseshoe crabs* Carcinoscorpius rotundicauda* [50,51], blue ring octopuses *H. maculosa* [52] and *H. lunulata* [44], and frogs *A. chiriquiensis* [53]. The foremost ecological role suggested for TTX residing in the egg masses and offspring is for protection. A recent study by Itoi* et al.* [45] demonstrates this possibility by showing that several predatory fish species ingested toxic pufferfish (*T. rubripes* and *T. niphobles*) larvae, but promptly spat them out. Immunohistological techniques revealed the localization of TTX in the outer layer of larvae of both pufferfish species. Several other studies have also shown the presence of TTX in pufferfish eggs and gonads signaling a potential role for the toxin in progeny protection [54,55].

**Table 1:** Tetrodotoxin (TTX) concentrations of egg masses and hatched larvae of *Pleurobranchaea maculata* (P.M.) collected from Pilot Bay, New Zealand on 9 September 2013 (*n* = 1) and average TTX concentrations in egg masses of *Stylochoplana* sp. (S.S.) kept in aquaria (*n* = 2).

Sample	TTX
P.M egg mass (−eggs)	ND
P.M. egg mass (+eggs)	3.7 mg·kg^−1^
P.M. larvae	48.3 pg·indivdual^−1^
S.S. egg masses	108 ± 2 mg·kg^−1^

## 3. Experimental Section

### 3.1. Specimen Collection

Toxic *P. maculata* and *Stylochoplana* sp. specimens were collected from Pilot Bay, Tauranga, New Zealand (37°63′5″ S, 176°17′6″ E). *Pleurobranchaea maculata* individuals were collected on 27 September 2012 and *Stylochoplana* sp. specimens on 12 September and 25 October 2013. Non-toxic *P. maculata* specimens were collected from Tasman Bay, New Zealand (41°05′ S, 173°06′ E) on 7 August 2012.

Egg masses from *P. maculata* were collected on 9 September 2013 from Pilot Bay and kept in an aerated aquarium for 3 days before freezing (−20 °C) sections for TTX analysis. Egg masses were tested in their entirety and small sections were carefully scraped clean of egg capsules leaving only the gelatinous casing for testing. The remaining egg masses were left in the aquarium until hatching (day 7). A sample of larvae (50 mL) was centrifuged (3000× *g*, 10 min), seawater removed, and frozen (−20 °C) for TTX analysis. Additional subsamples (50 mL) of larvae were collected, fixed with ethanol, and used for enumeration to determine the number of individuals in each sample. Counts were conducted using a 5 mL chamber and a dissecting microscope (Olympus SZ60).

Twenty-three* Stylochoplana* sp. specimens were collected from Pilot Bay (7 June 2012) and transported to laboratory aquaria in individual small plastic containers with 50 mL of seawater. Specimens were maintained in aerated aquariums (19 L) with 14 L of filtered seawater (0.22 μm). One individual laid two egg masses fourteen days after collection. Egg masses were removed from tanks and frozen (−20 °C) for TTX analysis. 

### 3.2. Histochemistry

*Pleurobranchea maculata* were aseptically dissected. Small sections of the mantle tissue from *P. maculata* were removed, while the gonad and digestive tissues were kept intact due to the fragility of these tissues. *Stylochoplana* sp. specimens were left whole because of their small size (ave. 60 mg) and fixed flat using the techniques described in Newman and Cannon [56]. Briefly, *Stylochoplana* sp. specimens were transferred using a small artist brush to a piece of filter paper dampened with ambient seawater in order to encourage them to lie flat. Filter paper was then transferred into a container with frozen fixative (2% glutaraldehyde/4% paraformaldehyde), which was left to melt.

Tissues and specimens were fixed overnight in 2% glutaraldehyde/4% paraformaldehyde, dehydrated through increasing concentrations of ethanol to xylene, embedded in paraffin, and sectioned at 7 to 10 μm thickness on a microtome (Leica RM 2055, Leica Biosystems, Wetzlar, Germany). Immunohistological sections were deparaffinized and rehydrated in ethanol before treatment with 3% H_2_O_2_/10% methanol to remove endogenous peroxidase activity followed by incubation with normal goat serum (VectorLabs, Burlingame, CA, USA) to prevent non-specific binding. Both the H_2_O_2_/methanol mixture and normal goat serum were diluted with 1× phosphate buffered saline (1× PBS, pH 7.2). Slides were then incubated with a TTX-specific monoclonal antibody (mAb) T20G10 diluted to 0.5 ugµmL^−1^[30] in concert with VECTASTAIN^®^ ABC kit (VectorLabs, Burlingame, CA, USA) according to the manufacturer’s instructions (Table 2). Visualization of the antigen-antibody complex was conducted using 3,3′-diaminobenzidine (DAB) substrate solution resulting in a brown color deposit. Sections were counterstained with Gill’s II Hematoxylin (Surgipath^®^, Leica Biosystems, Wetzlar, Germany), mounted, and observed under a light microscope (Leica DMRE with plan fluorite lenses, Leica Biosystems, Wetzlar, Germany).

**Table 2:** Immunohistological incubation scheme. Steps were undertaken at room temperature unless otherwise specified. PBS = phosphate buffered saline, mAB = monoclonal antibody, DAB = 3,3′-diaminobenzidine.

Step	Solution	Time (min)
1.	3% H_2_O_2_/10% methanol	10
2.	1× PBS	10 × 3
3.	Normal Goat Serum (Vector Labs)	20
4.	1× PBS	10 × 3
5.	mAB T20G10 *	Overnight at 4 °C
6.	1× PBS	10 × 3
7.	Biotinylated secondary antibody (anti-rabbit IgG) *	60
8.	1× PBS	10 × 3
9.	VECTASTAIN^®^ ABC reagent *	60
10.	1× PBS	10 × 3
11.	DAB	2–5
12.	Deionized H_2_O	5
13.	Counterstain (Gill’s II Hematoxylin)	2

* reagents diluted with 1× PBS, pH 7.2, modified with 0.5% Triton X-100 and 0.25% m/v type B gelatin.

Some sections of *Stylochoplana* sp. and *P. maculata* mantle tissue were also stained with Gill’s II hematoxylin (Surgipath^®^; Leica Biosystems, Wetzlar, Germany) and eosin (Surgipath^®^; Leica Biosystems, Wetzlar, Germany), and the Alcian Blue-Periodic Acid-Schiff (AB-PAS) method to differentiate between neutral and acidic mucins [57]. For AB-PAS staining, paraffin sections were rehydrated, stained with alcian blue (5 min), rinsed with distilled water, flooded with 1% periodic acid (2 min) and rinsed again. Slides were then immersed in Schiff’s reagent (8 min) and washed in running water (10 min). Sections were lightly counterstained with Mayer’s hematoxylin (2 min) before a final rinse with water. Sections were then dehydrated in an ascending ethanol series, cleared in xylene, mounted in D.P.X (Merck Millipore, Billerica, MA, USA), and observed under a light microscope (Leica DMRE with plan fluorite lenses, Leica Biosystems, Wetzlar, Germany).

### 3.3. Tetrodotoxin Analysis

Entire egg masses of *Stylochoplana* sp., specimens and sub-samples of egg masses from *P. maculata*, both with and without eggs, and hatched larvae were extracted for TTX. Samples (*ca.* 0.1 g) were first diluted 1:10 (w:v) with Milli-Q containing 0.1% v/v acetic acid. Each sample was manually homogenized with a glass pestle and vortexed to ensure complete disruption of tissues. Samples were centrifuged (3000× *g*, 10 min) and an aliquot of the supernatant was removed. This was diluted 1:10 with 100% methanol containing 0.1% v/v acetic acid and frozen (−20 °C) for at least 1 h. Samples were then centrifuged (3000× *g*, 10 min) and diluted 1:4 with 100% methanol containing 0.1% v/v acetic acid and analyzed for TTX using LC-MS as described in McNabb* et al.* [27].

## 4. Conclusions

Tetrodotoxin was found to be sequestered in the mantle, reproductive tissues, eggs, and larvae of *P. maculata*. Definitive characterization of the type of TTX-containing cells in the mantle of *P. maculata* is difficult to ascertain with paraffin techniques, and electron microscopy would greatly aid in identifying cell types and elucidating their potential functions. Tetrodotoxin localization in the digestive tissue could be indicative of a dietary source of TTX in this species. The *de novo* synthesis or sequestration of secondary metabolites from prey for use as a defense mechanism in Opisthobranchs is a well-known phenomenon (reviewed in [31]). The sequestration of TTX in the mantle, eggs, and larvae may be suggestive of a defensive role in *P. maculata*.

Localization of TTX in the pharynx, ova, and egg masses of *Stylochoplana* sp. could be indicative of ecological roles including aiding in capturing prey and protection of offspring, and further studies are required.

The methods by which *P. maculata* and *Stylochoplana* sp. sequester TTX are unknown. Immunohistologically-stained sections of *P. maculata* show that low concentrations of TTX are present throughout most tissues, while TTX is exclusively localized in the ova and pharynx of the *Stylochoplana* sp. This could be a product of differing anatomy (coelomate verses acoelomate) or that sequestration techniques differ between the two species. Tetrodotoxin-binding proteins have been isolated from a number of invertebrates including horseshoe crabs [58], xanthid crabs [59], and several gastropods [60]. Determining if these proteins are present in *P. maculata* or *Stylochoplana* sp. would assist in understanding the transfer and transport of TTX in these organisms.
